# Metagenomic analysis of microbial community structure and function in a improved biofilter with odorous gases

**DOI:** 10.1038/s41598-022-05858-9

**Published:** 2022-02-02

**Authors:** Jianguo Ni, Huayun Yang, Liqing Chen, Jiadong Xu, Liangwei Zheng, Guojian Xie, Chenjia Shen, Weidong Li, Qi Liu

**Affiliations:** 1 College of Life and Environmental Science, Hangzhou Normal University, Hangzhou, 310036 Zhejiang People’s Republic of China; 2 Hangzhou Ecological Environment Bureau of Xiaoshan Branch, Hangzhou, 311201 Zhejiang People’s Republic of China; 3 College of Qianjiang, Hangzhou Normal University, Hangzhou, 310036 Zhejiang People’s Republic of China; 4 Taizhou Pollution Prevention and Control Engineering Technology Center, Taizhou, 318000 Zhejiang People’s Republic of China; 5 Taizhou Ecological Environment Bureau of Linhai Branch, Taizhou, 317000 Zhejiang People’s Republic of China

**Keywords:** Biotechnology, Computational biology and bioinformatics, Environmental sciences

## Abstract

Biofilters have been broadly applied to degrade the odorous gases from industrial emissions. A industrial scale biofilter was set up to treat the odorous gases. To explore biofilter potentials, the microbial community structure and function must be well defined. Using of improved biofilter, the differences in microbial community structures and functions in biofilters before and after treatment were investigated by metagenomic analysis. Odorous gases have the potential to alter the microbial community structure in the sludge of biofilter. A total of 90,016 genes assigned into various functional metabolic pathways were identified. In the improved biofilter, the dominant phyla were *Proteobacteria*, *Planctomycetes*, and *Chloroflexi*, and the dominant genera were *Thioalkalivibrio*, *Thauera*, and *Pseudomonas*. Several xenobiotic biodegradation-related pathways showed significant changes during the treatment process. Compared with the original biofilter, *Thermotogae* and *Crenarchaeota* phyla were significantly enriched in the improved biofilter, suggesting their important role in nitrogen-fixing. Furthermore, several nitrogen metabolic pathway-related genes, such as *nirA* and *nifA*, and sulfur metabolic pathway-related genes, such as *fccB* and *phsA*, were considered to be efficient genes that were involved in removing odorous gases. Our findings can be used for improving the efficiency of biofilter and helping the industrial enterprises to reduce the emission of waste gases.

## Introduction

Odorous gases are typical outdoor air pollutants that have effects on the environment and health of human beings^1,2^. With the development of economy, odorous gases from industrial emissions become major factor to haze contamination in the world^3^. As a public health concern, odorous gases is a significant threat to personal health and comfort^4,5^. The major components of odorous gases are nitrogen containing compounds, such as NH_3_ and NO_x_, sulfur containing compounds, such as H_2_S and SO_2_, and volatile organic compounds (VOCs), such as non methane total hydrocarbons, oxygenated organic compounds, and halogenated hydrocarbons^6,7^. Most of these nitrogen- and sulfur-containing compounds have pungent odor. In addition to strong offensive smell, odorous gas need to be solved due to their long-term health effects^8^. Thus, controlling odorous gas emissions is essential for reducing air pollution.

In past years, a number of technologies have been developed to control air pollution caused by odorous gases. Most of these technologies can be classified into three different groups, including physical methods, chemical methods, and biological methods^9^. Although physical–chemical methods have high efficiency to treat odorous gases, several adverse factors, such as high cost, high concentration and secondary pollution, greatly limit their applications^10^. Biological technologies are recently considered to be environment friendly methods for removing odorous gases, compared with physical–chemical methods^11^. Three major categories, including biofilter, bioscrubbers, and biotrickling filters, were widely applied as biological technologies^9^.

Biofilter is commonly used for the harmlessness treatment of industrial odorous gases by forcing waste gases to rise through a layer of packed material^12^. Several key parameters, such as nutrients, temperature, pH value, and microbial community, determined the efficiency of the biofilter^13^. Packing materials in the biofilters contain specific microorganisms, which are the key characteristics and largely varied within different biofilters treating different pollutants^14^. A greatly effective microbial community plays an important role in degrading pollutants into harmless small molecules^15^. It is therefore important to take the improvement of microbial community into consideration.

Studies on microbial community and function of biofilter packing is a shortcut to optimize the management of biofilter system^16^. Most previous works have focused on the isolation and identification of high efficient microbial species in the packed material of biofilter system^17^. For example, an *Acidithiobacillus* and a *Thiobacillus* species were considered to play a dominant role in novel horizontal flow biofilm reactors under H_2_S treatment^18^. The functions of *Sphingomonas* sp. in ethylbenzene degradation, *Thiomonas* sp. in carbon disulfide degradation, and *Acidithiobacillus* sp. in hydrogen sulfide degradation have been well uncovered^19–21^. With the application of high-throughput sequencing, the traditional methods are thought to be very inefficient.

High-throughput studies have focused on the structural and functional responses of microbial communities during the treatment of contaminants^17,22,23^. Previous analysis showed that there are great differences in microbial communities and structures between high N_2_O emission area and low N_2_O emission area^24^. In anaerobic ammonium oxidation systems, high-throughput sequencing results revealed that *Kuenenia* was the dominant species of anammox bacteria^25^. In addition to microbes, a number of functional genes were considered to play important roles in metabolism of nitrogen- and sulfur-containing compounds. For examples, *nirK* and *nirS* were treated as maker genes of bacterial community under the nitrous oxide treatment^25^. Bacterial ammonia monooxygenase (*amoA*) gene was applied to evaluate the bacterial communities in different types of biofiltration technologies^26^. Although many efficient bacteria and functional genes have been identified, effects of odorous gases on the microbial community and structure in biofilter system are largely unknown.

Our previous study have discovered the shifts in microbial communities and structures in a commercial scale biofilter^27^. After a year of adjustment, an improved biofilter showed more efficient role in assimilating H_2_S and NH_3._ In the present study, metagenomics sequencing was used to screen novel microbial species and functional genes involved in degradation of odorous gases. Comparison of the original and improved biofilters will provide useful targets for improving the efficiency of biofilter.

## Materials and methods

### Materials and sampling

Waste gas treatment plant used in the present study is the same to our previous study^27^. In briefly, the treatment plant was filled with mixed media (activated carbon, wheat bran, and sawdust at 1:1:2). The waste gas passed through the media in an up-flow direction. Sample materials were isolated from improved sludge in this equipment after a 3-week acclimation period with clean airflow, followed by a 2-week period of odor contaminated airflow. The sample isolated before the treatment of clean airflow was named as control sample (CS) and the sample isolated after the treatment of odorous gases was named as treated sample (TS). The main components of odorous gases are H_2_S and NH_3._ For H_2_S, the initial concentration is 19.2 mg m^−3^ and the emission rate is about 0.2 kg h^−1^. For NH_3_, the initial concentration is 9.3 mg m^−3^ and the emission rate is about 0.2 kg h^−1^.

### Determination of NH_3_ and H_2_S concentrations

The NH_3_ concentration was determined using the Nessler’s reagent colorimetry method^28^. In detial, Nessler reagent was purchased from Sigma-Aldrich with product No. 72190. The NH_3_ and H_2_S gasses were collected by an integrated air collector (Tuowei Instrument Ltd, Qingdao, China). NH_3_ in the air was absorbed using 0.05 mol/L dilute H_2_SO_4_. The NH_4_ + ions react with the Nessler reagent to form a yellow–brown complex. The absorbance of the complex proportional to the NH_3_ concentration was determined at a wavelength of 420 nm. The H_2_S concentration was determined using the methylene blue spectrophometry method (GB/T11742-89, China). The absorbance was measured at a wavelength of 665 nm. The minimum detectable concentration was 0.001 mg/m^3^. The concentration of H_2_S was calculated according to formula that was published in a previous work^29^.

### DNA isolation and library construction

Microbial genomic DNA was extracted using a Omega DNA kit (D4015-02) following the producer’s procedures. Then, the DNA samples were purified by 1% agarose gel electrophoresis. The quality of DNA samples was analyzed by a NanoDrop spectrophotometer according to the criterion of A260/A280 between 1.7 and 1.8 and A260/A230>1.7. Afterwards, DNA samples were cut into small fragments of about 250 bp to construct paired-end sequencing libraries. DNA templates were then processed using the TruseqTM kit according to the manufacture’s instruction.

### Illumina sequencing and raw data uploading

High-throughput sequencing was processed on a HiSeq4000 platform and the mode was set at PE150. The metagenomic sequence reads were processed to remove the invalid reads. Low quality reads, including adapters, taqs and N > 5% reads, were removed using softwares Cutadapt v1.9 and Fqtrim v0.94 with sliding-window algorithm. The sequences without sequencing tags and adapters were subjected to quality control using the Galaxy FastX software with a minimum size 100 bp and minimum quality score 20. The raw sequence data has been submitted to the NCBI as a BioProject with accession number PRJNA699130 (Sludge 1–10: CS1–10 and Sludge 11–12: TS1–10).

### Sequence assembly and unigene identification

To get the metagenomic contigs, de novo assembly was performed using the clean reads. For taxonomic affiliations and functional annotations, generated metagenomic contigs larger than 500 bp in length were subjected to the MG-RAST server with related metadata files. The result sequences were used to predict their protein coding sequences (CDS) using MetaGeneMark v3.26 software. Then, the CDSs were clustered by CD0HIT v4.6.1 software to produce unigene, which is a widely used gene database and a non-redundant gene database formed by the collection of the same loci by computer. The functional annotation of unigenes was performed by the Reduced Alphabet based Protein similarity Search tool against the Nr, Gene Onotology (GO), Kyoto Encyclopedia of Genes and Genomes (KEGG)^30^, and Carbohydrate-Active enzymes (CAZy) databases with default parameters.

### Taxonomic profile analysis

For species classification, all unigenes were searched against the Nr_meta database, a sub-database of Nr database, using the DIAMOND software with evalue < 1e^−5^. Different species classification levels, including Phylum, Class, Order, and Family, were analyzed using the NCBI Taxonomy system with a Lowest Common Ancestors (LCA) algorithm. A lineage without classified information in the database was set as ‘unclassified’. We classified the microorganisms with a relative abundance lower than 0.10% as ‘others’.

### GO and KEGG enrichment analysis

For each GO or KEGG category, a two-tailed Mann–Whitney U exact test was employed to describe the enrichment or out-competed of potential genes and pathways in different comparisons. Correction for multiple hypothesis testing was carried out using standard false discovery rate (FDR) control methods. The GO or KEGG term with a corrected *P* value < 0.05 is considered significant. The MeV software was used to visualize the enrichment results. The “rich factor” refers to the ratio of the genes located in the GO entry to the total number of genes located in the GO entry in all annotated genes. The rich factor indicated the degree of enrichment.

### QRT-PCR validation of several selected genes

The total DNA were extracted using a Omega DNA kit (D4015-02) following the producer’s procedures. The fragment of each selected gene was amplified using PCR method. The PCR product was purified and used as the standard. Then, a series of purified DNA products was diluted gradient with known copy number. Fluorescent quantitative PCR was used to amplify the selected gene, and the standard curves of copy number and cycle number were made. According to the standard curve, the copy number of the selected gene in different treatment groups was calculated. SYBR Premix Ex Taq Kit (TaKaRa, Dalian, China) and a DNA Sequence Detection System (ABI PRIM 7700) were used for the RT-PCR experiment. Independent DNA samples from CS and TS in the improved biofilter and the original version were used for the RT-PCR experiment. The copy number analysis was performed for three biological replications. The primer sequences were listed in Table S1.

### Statistical analysis

Three parallel experiments were carried out for sequencing. All data on the diversity indexes, the relative abundances of unigenes, and the H_2_S and NH_3_ levels were processed using SPSS 18.0. One-way analysis of variance (ANOVA) was applied to analyze the differences between the CS and TS sample groups. Mann–Whitney U test were conducted to detect differences in microbial community structures between two groups. The *P* value was produced by the false discovery rate (FDR) analysis and adjusted using the Benjamini and Hochberg’s method^31^. A significant difference was indicated by a probability value (*P*) less than 0.05.

## Results

### Improved removal performance for NH_3_ and H_2_S

Our study evaluated the improved performance in removing odorous gases containing NH_3_ and H_2_S. After treatment, the emission concentrations of H_2_S decreased from 35.3 to 0.32 mg m^−3^ in the original version and to 0.14 mg m^−3^ in the improved version; the emission rate of H_2_S were decreased from 0.26 to 0.0032 kg h^−1^ in the original version and to 0.0019 kg h^−1^ in the improved version. After treatment, the emission concentrations of NH_3_ decreased from 16.1 to 2.2 mg m^−3^ in original version and to 1.1 mg m^−3^ in the improved version; the emission rate of NH_3_ were decreased from 0.12 to 0.022 kg h^−1^ in the original version and to 0.009 kg h^−1^ in improved version (Table S2).

### Detail information of the metagenomes

A total of 6.18E+08 and 5.53E+08 raw reads were obtained from the CS and TS samples, respectively. Then, 5.90E+08 clean reads from CS and 5.30E+08 clean reads from TS were used to assemble metagenomes (Table S3). After filtering out the low quality sequences, 143,441 contigs (N50: 1689 bp) were obtained from the CS sample and 155,721 contigs (N50: 1476 bp) were obtained from the TS sample (Fig. S1). To get an overview of the metagenomic variations, a PCA was performed, and the percentages of explained value in the analysis of PC1 and PC2 were 99.12% and 0.44%, respectively (Fig. S1). Correlation coefficient analysis indicated the sequencing data had good repeatability (Fig. S2).

### Functional prediction and classification of unigenes

Based on the assembled contigs, a total of 901,016 unigenes with an average length of 660 bp and GC content of 58% were predicted. All the unigenes were detected in the CS sample and only 875,737 unigenes were detected in the TS sample. Length distribution of all predicted unigenes was showed in Fig. S3 and read counts of unigenes were showed in Fig. S4.

In total, 726,022 unigenes were assigned to different functional KEGG pathways. In the ‘metabolism’ category, ‘carbohydrate metabolism’ (59,872 unigenes), ‘amino acid metabolism’ (49,584 unigenes), and ‘cofactors and vitamins metabolism’ (30,315 unigenes) are the most typical KEGG pathways. In the ‘genetic information processing’ category, most unigenes were classed into the ‘translation’ (18,585 unigenes), ‘replication and repair’ (15,850 unigenes), and ‘folding, sorting and degradation’ (11,594 unigenes) pathways. In the ‘environmental information processing’ category, most unigenes were grouped into the ‘signal transduction’ (27,557 unigenes) and ‘membrane transport’ (26,101 unigenes). In the ‘cellular processes’ category, the major terms were ‘cell motility’ (19,559 unigenes) and ‘cellular community’ (9011 unigenes) (Fig. S5).

### Taxonomic profile of the two metagenomes

Based on the predicted open reading frames (ORFs), the taxonomy annotation and abundance of microbial species derived from the two sample groups was analyzed. At phylum level, 184 taxa were summarized from the two sample groups (Table S4). According to their annotation, *Proteobacteria* (37.7%), *Planctomycetes* (8.7%), *Chloroflexi* (2.5%), *Bacteroidetes* (2.2%), *Cyanobacteria* (1.6%), and *Actinobacteria* (1.0%) were considered to be the dominant phyla. At the phyla level, *Methanosarcinaceae* and *Ichthyobacteriaceae* are significantly enriched, and *Morchellaceae*, *Nautiliaceae* and *Sterolibacteriaceae* are significantly out-competed.

**Figure 1:**
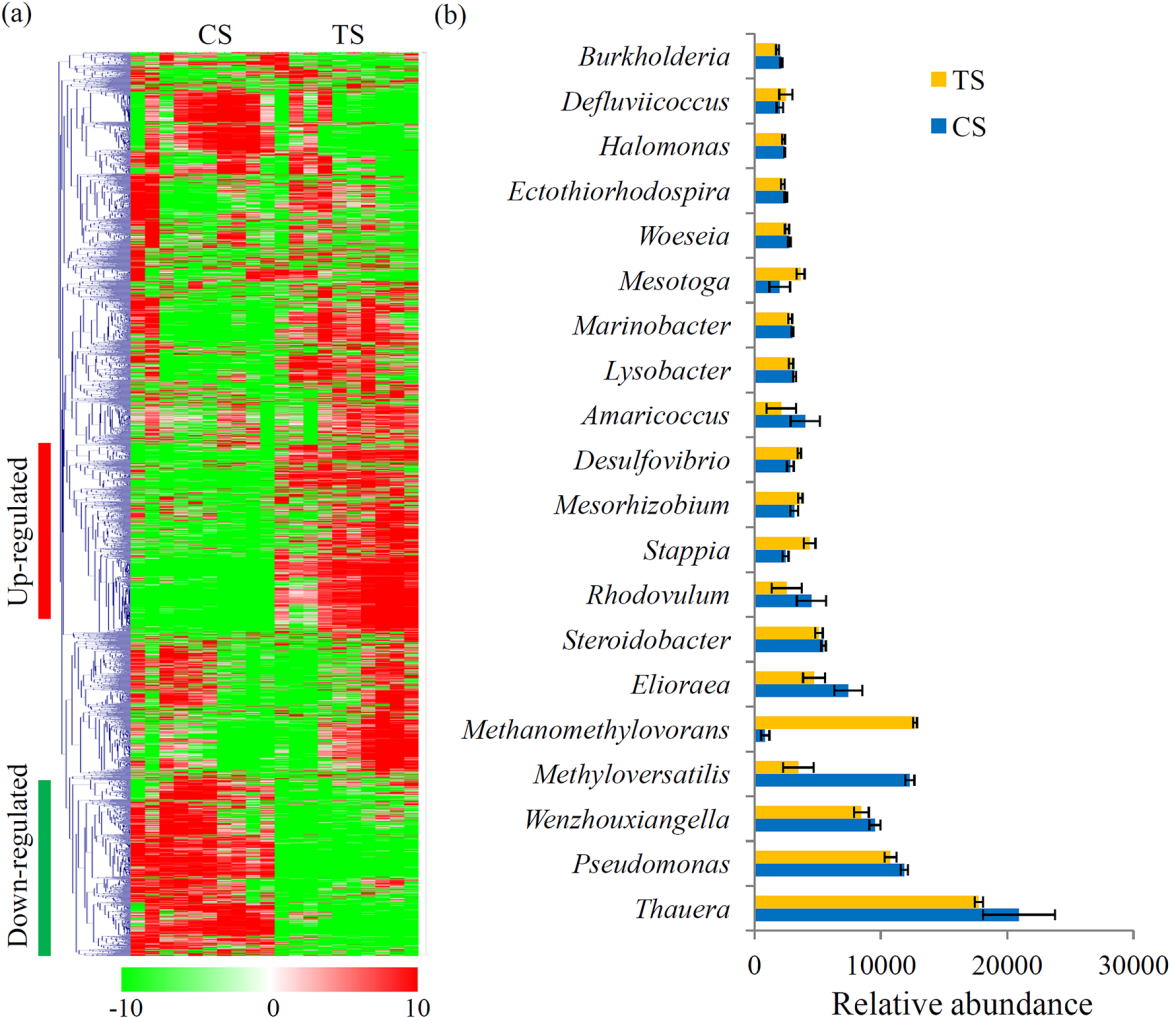
Comparative taxonomic profile of CS and TS metagenomes. (**a**) Microbial compositions for both CS and TS sample groups at genus level. Color intensity in each panel shows the relative abundances of each representative genus in the CS and TS sample groups. The heatmap scale ranges from − 10 to + 10 on a log_2_ scale. (**b**) Microbial compositions for both CS and TS sample groups at genus level.

At the genus level, a total of 3619 genera were obtained from the two sample groups. Among these genera, there are 112 genera with a relative abundance more than 0.1% of the total microbes. Microbial compositions for both CS and TS sample groups at genus level were showed in Fig. 1a. According to their annotation, *Thauera* (1.94%), *Pseudomonas* (1.13%), and *Wenzhouxiangella* (0.89%) were considered to be the dominant genera (Fig. 1b). Several dominant genera, including *Methanomethylovorans*, *Stappia*, *Mesorhizobium*, *Desulfovibrio*, *Mesotoga*, and *Defluviicoccus*, were significantly enriched during the treatment process. Contrarily, several other dominant genera, such as *Methyloversatilis*, *Elioraea*, *Rhodovulum*, and *Amaricoccus*, were significantly out-competed during the treatment process (Table S5).

### Analysis of the enrichment of genes during the treatment

**Figure 2:**
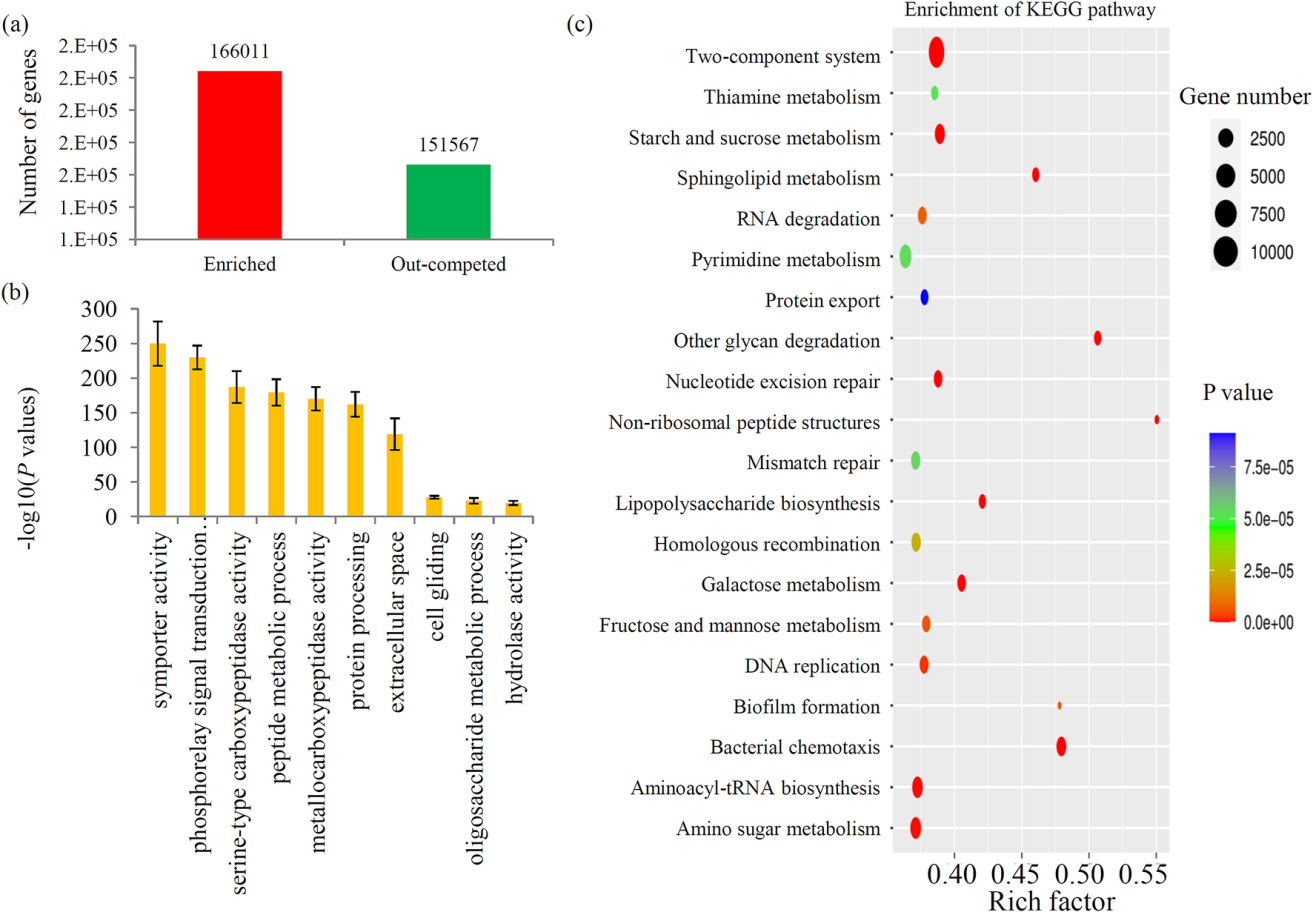
Enrichment analysis of the genes during the treatment. (**a**) The number of enriched and out-competed genes after treatment. (**b**) GO significance analysis of the differential genes between the CS and TS sample. (**c**) KEGG significance analysis of the differential genes between the CS and TS sample.

A large number of genes, including 166,011 enriched and 151,567 out-competed genes, were identified in our study (Fig. 2a). According to their annotations, most of the differential genes were assigned into different categories. For the GO classification, the top five significant enriched GO terms were ‘symporter activity’, ‘phosphorelay signal transduction system’, ‘serine-type carboxypeptidase activity’, ‘peptide metabolic process’, and ‘metallocarboxypeptidase activity’ (Fig. 2b). Most of the differential genes were grouped into 187 KEGG metabolic pathways (Table S6). Based on their KEGG classification, the top five significant enriched KEGG terms were ‘two-component system’, ‘other glycan degradation’, ‘sphingolipid metabolism’, ‘galactose metabolism’, and ‘bacterial chemotaxis’ (Fig. 2c).

### Analysis of xenobiotic biodegradation pathway-related KEGGs

**Figure 3:**
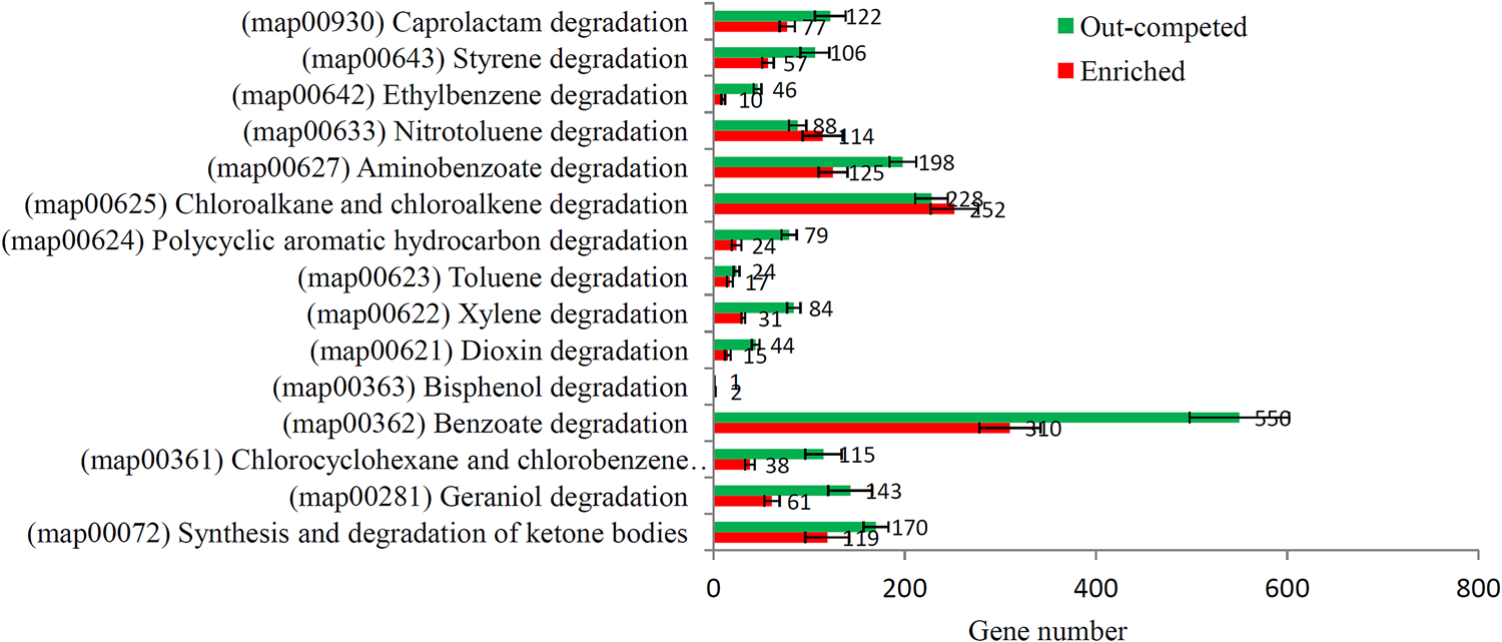
Analysis of xenobiotic biodegradation pathway-related KEGG terms. The number of enriched and out-competed genes related to xenobiotic biodegradation pathways after treatment.

Previous studies have reported several xenobiotic biodegradation pathways in different microbes^32^. In our study, 15 enriched xenobiotic biodegradation pathways were identified (Table S7). The benzoate degradation pathway (map00363) contained the largest number of genes, including 310 enriched and 550 out-competed genes. The second largest xenobiotic biodegradation pathway was the chloroalkane and chloroalkene degradation pathway, including 252 enriched and 228 out-competed genes. Aminobenzoate degradation pathway was the third largest xenobiotic biodegradation pathway, containing 125 enriched and 198 out-competed genes (Fig. 3).

### Analysis of the genes involved in the nitrogen metabolic and sulfur metabolic pathways

**Figure 4:**
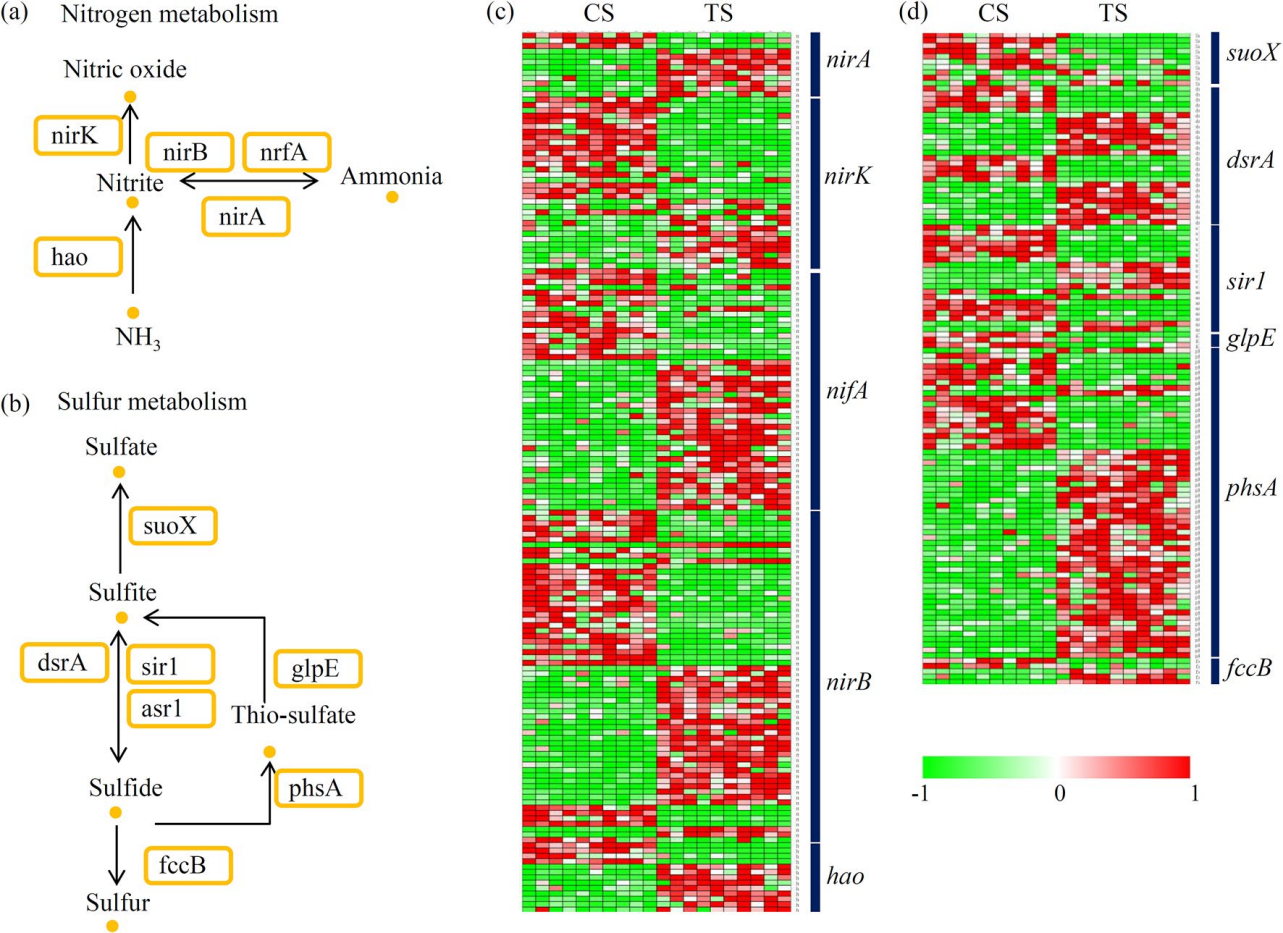
Analysis of the genes involved in the nitrogen metabolic and sulfur metabolic pathways. (**a**) Overview of the nitrogen metabolic pathway. (**b**) Overview of the sulfur metabolic pathway. (**c**) The relative abundances of the genes involved in the nitrogen metabolic pathway. (**b**) The relative abundances of the genes involved in the sulfur metabolic pathway. Red indicated low abundance and green indicated high abundance of each gene. The heatmap scale ranges from − 1 to + 1 on a log_2_ scale.

A number of genes involved in the nitrogen metabolic and sulfur metabolic pathways have been reported in the past years^33^. For the nitrogen metabolism, the genes, including *nirK*, *nirB*, *nrfA*, *hao*, and *nirA*, were identified in our study (Fig. 4a). For the sulfur metabolism, seven key enzymes encoding genes, including *suoX*, *dsrA*, *sir1*, *asr1*, *glpE*, *phsA*, and *fccB*, were identified in our study (Fig. 4b). For the nitrogen metabolic pathway, most of the *nirA* and *hao* genes were significantly enriched during the treatment (Fig. 4c). For the sulfur metabolic pathway, most of the *phsA* genes were enriched and most of the *suoX* genes were out-competed during the treatment (Fig. 4d).

### Comparison of the microbial community between original and improved biofilters

**Figure 5:**
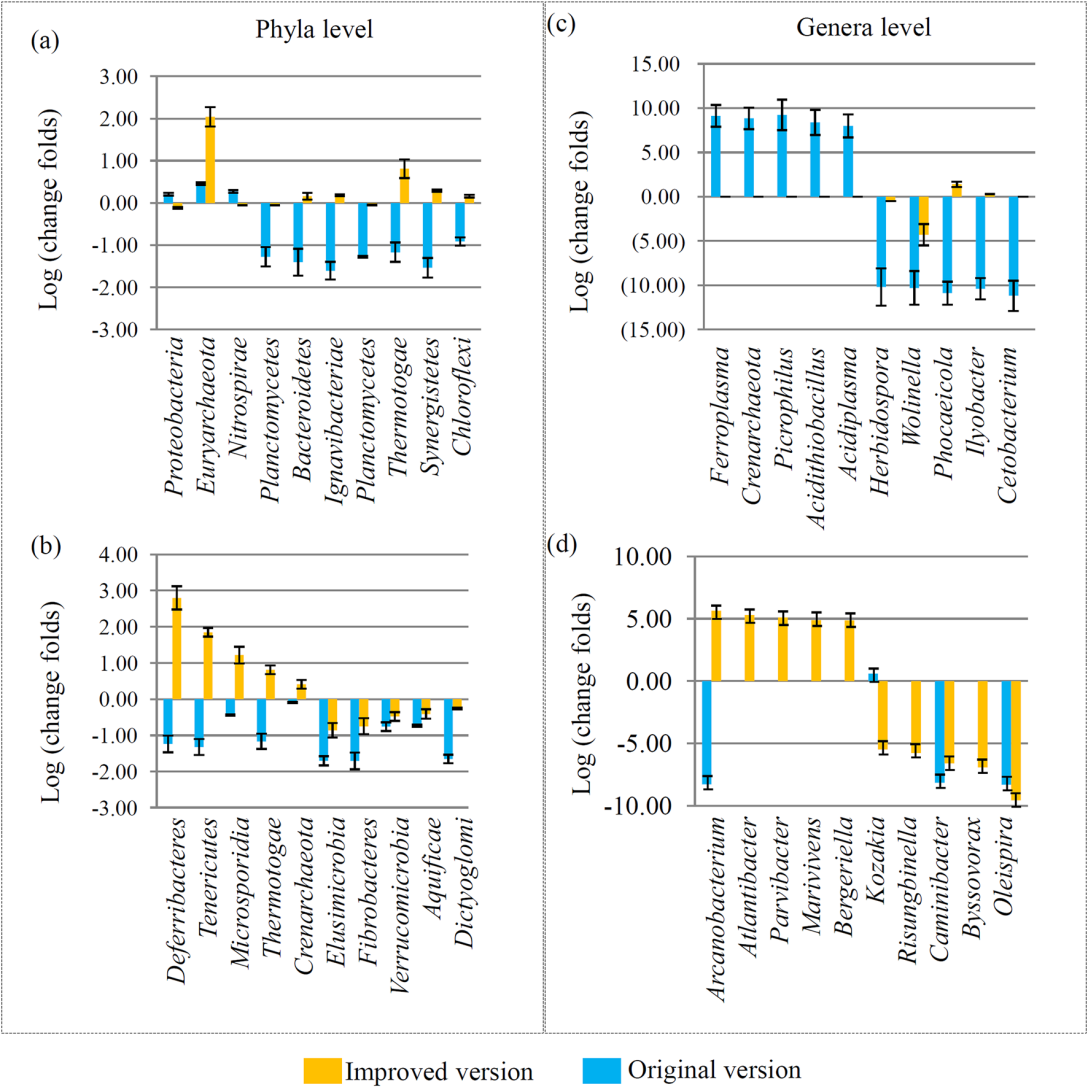
Comparison of the microbial community between original and improved biofilters. (**a**) Changes in microbial communities of the top 10 phyla that were significantly changed in the original biofilter. (**b**) Changes in microbial communities of the top 10 phyla that were significantly changed in the improved biofilter. (**c**) Changes in microbial communities of the top 10 genera that were significantly changed in the original biofilter. (**d**) Changes in microbial communities of the top 10 phyla that were significantly changed in the improved biofilter.

In our study, comparison of the microbial community between the original and improved biofilters has been performed. Firstly, we analyzed the changes between the original and improved biofilters at phyla level. In the original biofilter, the significantly enriched phyla were *Proteobacteria*, *Euryarchaeota* and *Nitrospirae*, and the significantly out-competed phyla were *Ignavibacteriae*, *Bacteroidetes*, and *Planctomycetes* (Fig. 5a). In the improved biofilter, the significantly enriched phyla were *Deferribacteres*, *Tenericutes*, and *Microsporidia*, which showed opposite responses to the original version. While in the improved biofilter, the significantly out-competed phyla were *Elusimicrobia*, *Fibrobacteres*, and *Verrucomicrobia*, which showed similar responses to the original version (Fig. 5b).

The changes between original and improved biofilters at genera level were analyzed. In the original biofilter, the significantly changed genera were *Ferroplasma* and *Cetobacterium*, which showed no responses in the improved version (Fig. 5c). In the improved biofilter, the most enriched genera was *Arcanobacterium*, which was siginificantly out-competed in the original version, and the most out-competed genera was *Oleispira*, which showed similar response to the original version (Fig. 5d).

### Comparison of the functional genes between original and improved biofilters

**Figure 6:**
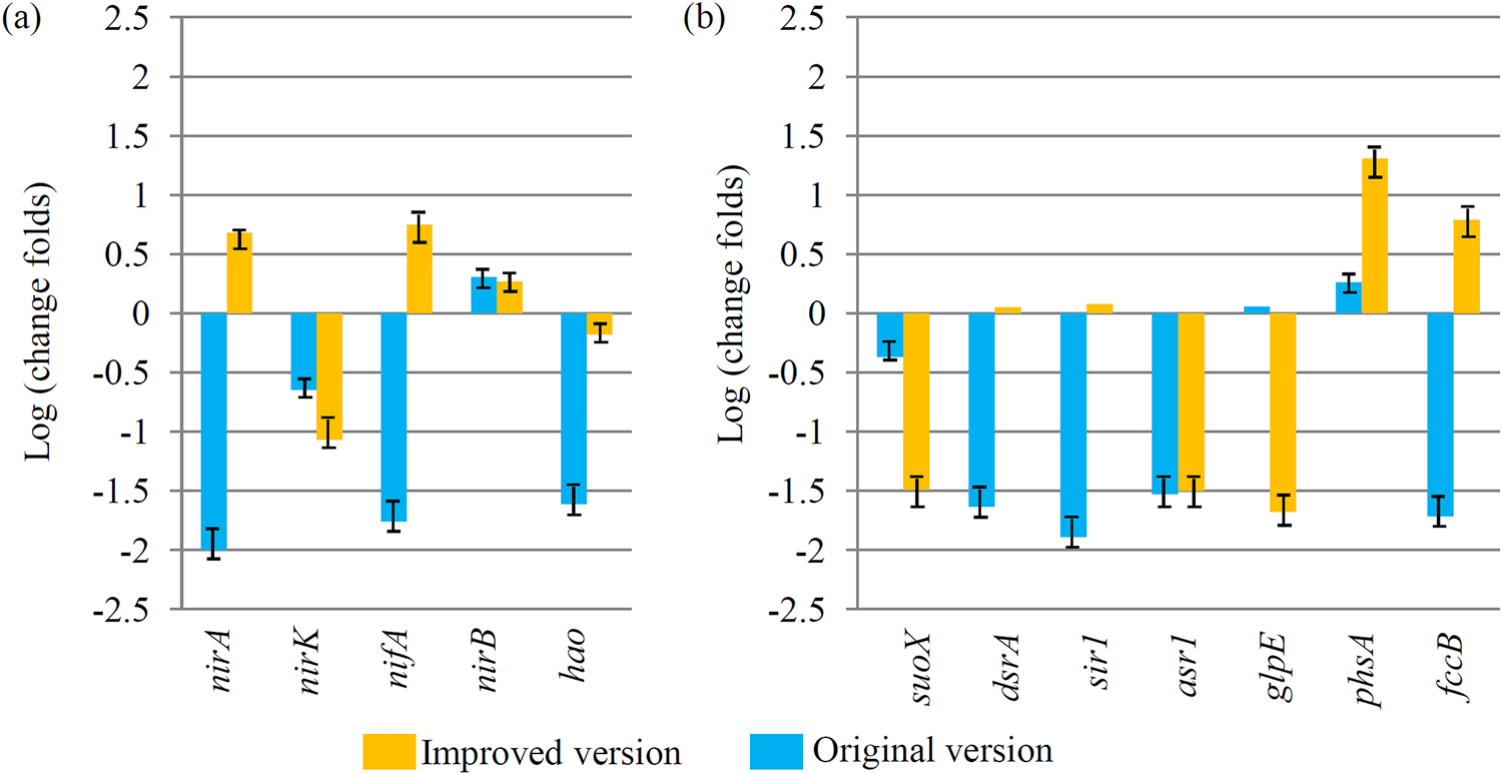
Comparison of the functional genes between original and improved biofilters. (**a**) The average expression levels of nitrogen metabolic pathway-related genes in both of the original and improved biofilters. (**b**) The average expression levels of sulfur metabolic pathway-related genes in both of the original and improved biofilters.

Comparison of the functional genes between original and improved biofilters has been also performed. For the nitrogen metabolic pathway, average expression levels of the *nirA*, *nifA* and *nirB* genes were significantly enriched in the improved biofilter and only the *nirB* genes were enriched in the original version (Fig. 6a). For the sulfur metabolic pathway, most of the key genes were enriched in both of two biofilters, except for *phsA* and *fccB*. The *phsA* and *fccB* genes were significantly enriched in the improved version (Fig. 6b).

### Validation of several key genes involved in the nitrogen and sulfur metabolic pathways

To confirm the gene expression changes, two nitrogen metabolic pathway-related genes (*nirA* and *nifA*) and two sulfur metabolic pathway-related genes (*phsA* and *fccB*) were selected and determined by qRT-PCR analysis. Our qRT-PCR results basically confirmed the metagenomic data (Figs. S6 and S7).

## Discussion

Microbial community structures were largely affected by odorous gas treatment^34,35^. Take advantage of a commercial scale biofiltration system, our previous study have revealed the changes in microbial communities and identified a number of functional genes involving in removing odorous gases^27^. Recently, the original biofiltration system has been improved and higher effective in treating odorous gases was achieved by extending the acclimation period. Our present study has been detected 901,016 genes, which was larger than the genes detected by our previous study (496,718 unigenes), giving us an opportunity to screen more microbes and functional genes involved in removing odorous gases.

*Proteobacteria* was the most abundant phylum in many different biofiltration systems during the treatment process. For examples, *Proteobacteria* occupies 58–92% share in two butyric acid biofiltration systems^17^, 3.55–51.03% share in a series of laboratory-scale landfill reactors^36^, and 51.9% share in a drinking water biofiltration^37^. Addition to *Proteobacteria*, *Planctomycetes* and *Chloroflexi* also were reported to be the dominant phyla in various biofilter reactors^38,39^. Our data also showed that *Proteobacteria*, *Planctomycetes* and *Chloroflexi* phylum possessed the largest shares in the improved biofilter, suggesting the consistency in matters of microbial community. Previous studies have pointed out that microbes belonging to *Proteobacteria* participated in blackening and odor formation processes of odorous gases^40^. During the treatment process, microbes belonging to *Proteobacteria* were significantly reduced, indicated a close relationship between *Proteobacteria* and odorous gas removal.

At the genera level, a number of microbes were reported to be involved in biological degradation. For examples, *Methanomethylovorans* was the dominant genus for degrading various types of polycyclic aromatic hydrocarbon^41^. For methanogenic degradation of tetraethylammonium hydroxide, *Methanomethylovorans* was the dominant genus that was isolated from the methanogenic degradation bioreactor^42^. *Mesorhizobium* was another important genus associated the emission of sulfur-containing odors such as hydrogen sulfide, methyl mercaptan and dimethyl disulfide^43^. *Desulfovibrio* sp. SB8 was reported to be served as core player for sulfate-reduction coupling polycyclic aromatic hydrocarbon degradation^44^. In our study, *Methanomethylovorans*, *Desulfovibrio* and *Mesorhizobium* were the most significantly up-regulated dominant genera during the treatment process, suggesting their important roles in removing odorous gases. Our study provided important guidance for identification of dominant microbes in odorous gas biofilter.

Elimination of NH_3_ and H_2_S is an important objective for the removal of odorous gases using biofilters^45^. Complex metabolic networks containing a number of key genes have been revealed in different microbes^46^. NH_4_^−^ ion assimilation involves a nitrogen-cycling consisted of several key genes^47^. Meanwhile, reaction of sulfur cycle that occurs in bio-system is essential for the elimination of sulfur-containing odorous gases^48^. In our study, a large number of homologous genes referring to each key gene were identified by metagenomic sequencing, giving us an opportunity to screen novel nitrogen-cycling microbial genes^47,49^. Significantly changes in relative abundance of nitrogen- and sulfur-cycling related genes indicated an important role of nitrogen and sulfur metabolisms in odorous gas removal.

Investigation of the differences between original and improved biofilters will help us to screen efficient microbes and functional genes. Analysis of microbial communities in Nakabusa hot springs indicated that several possible nitrogen-fixing bacteria were belonged to the phylum *Thermotogae*^50^. Another study has reported that *Crenarchaeota* is closely related to the ammonia-oxidizer '*Nitrosopumilus maritimus*', indicating its potential contribution to nitrification in the biofilter^51^. Our study showed that *Thermotogae* and *Crenarchaeota* phyla were significantly enriched in the improved biofilter, suggesting their important role in nitrogen-fixing. Nitrogen fixations generally inhibited in the presence of ammonia. A previous study has showed that *nifA-like* gene of *Azospirillum lipoferum* Br17 was expressed under conditions of nitrogen fixation and in the presence of ammonia^52^. It indicated that the nitrogen fixation can occur in the presence of ammonia in the medium within biofilters. A number of nitrite metabolism-related functional genes have been identified in the past years. For examples, several nitrite reductases encoded by *nirA*, *nirB* and *nirK* genes are important enzymes involved in fungal denitrification^53^. The nif-specific activator NifA was reported to be involved in the regulation of nitrogenase activity during the nitrogen fixation process^54^. The expression of *hao* gene was highly correlating nitrogen removal and N_2_O emission characteristics^55^. Compared with the original version, *nirA* and *nifA* genes were significantly enriched the improved biofilter, suggesting their roles in assimilating ammonia-containing odorous gases. In the sulfur metabolic pathway, flavocytochrome c sulfide dehydrogenase encoding gene *FccB* played a role in oxidization of endogenous or exogenous H_2_S^56^. *PhsA* encoding the putative thiosulfate reductase participated in an intraspecies sulfur cycle^57^. Both of *fccB* and *phsA* were significantly enriched in the improved biofilter, suggesting an activated oxidization of H_2_S during the odorous gas treatment.

Metagenomic sequencing provides an efficient means for us to screen microbial populations containing specific genes. Based on the screened microorganisms, increasing the enrichment of specific microbial populations in the bioreactor is helpful to increase the efficiency of the bioreactor. Therefore, our metagenomic information can be used for improving the efficiency of biofilter and helping the industrial enterprises to reduce the emission of waste gas.

## Supplementary Information

Supplementary Tables.

Supplementary Figures.
