# Supplementary material for: Metagenomic analysis of microbial community structure and function in a improved biofilter with odorous gases
Source: Sci Rep. 2022 Feb 2;12:1731. doi: 10.1038/s41598-022-05858-9 (PMC8810771; doi:10.1038/s41598-022-05858-9)
Supplement: Supplementary file 2 — Supplementary Figures. [file 41598_2022_5858_MOESM2_ESM.pdf]

Metagenomic analysis of microbial community structure and function in a improved biofilter with odorous gases

Jianguo Ni[1, 2, a], Huayun Yang[1, 3, a], Liqing Chen[1], Jiadong Xu[4], Guojian Xie[1], Chenjia Shen[1], Weidong Li[3], Qi Liu[1,*]

[1] College of Life and Environmental Science, Hangzhou Normal University, Hangzhou 310036, Zhejiang, People's Republic of China

[2] Hangzhou Ecological Environment Bureau of Xiaoshan Branch, Hangzhou, 311201, Zhejiang, People's Republic of China

[3] College of Qianjiang, Hangzhou Normal University, Hangzhou 310036, Zhejiang, People's Republic of China

[4] Taizhou Pollution Prevention and Control Engineering Technology Center, Taizhou 318000, Zhejiang, People's Republic of China

[a] These authors contributed equally to the article

[*] Correspondence: qiliu@hznu.edu.cn (Q. L.); Tel.: +86-571-28867258 (Q. L.); +86-571-28865333 (Q. L.)

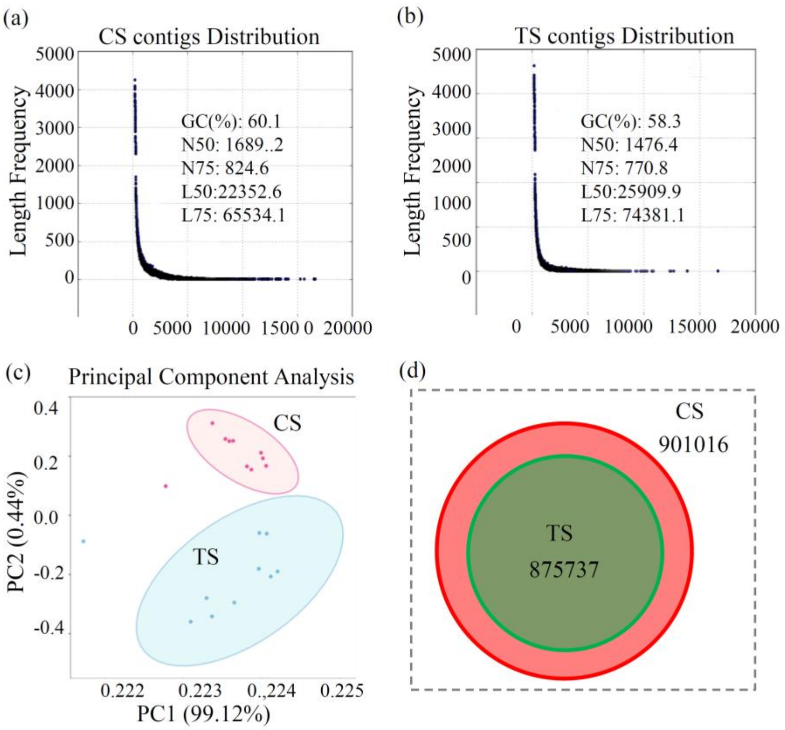

Figure S1 **Overview of the metagenomes.** (a) The detail information of all contigs from the sample group CS. (b) The detail information of all contigs from the sample group TS. (c) PC analysis of the data from two sample groups. (d) The numbers of unigene identified in the BT and AT sample groups are shown in a Venn diagram.

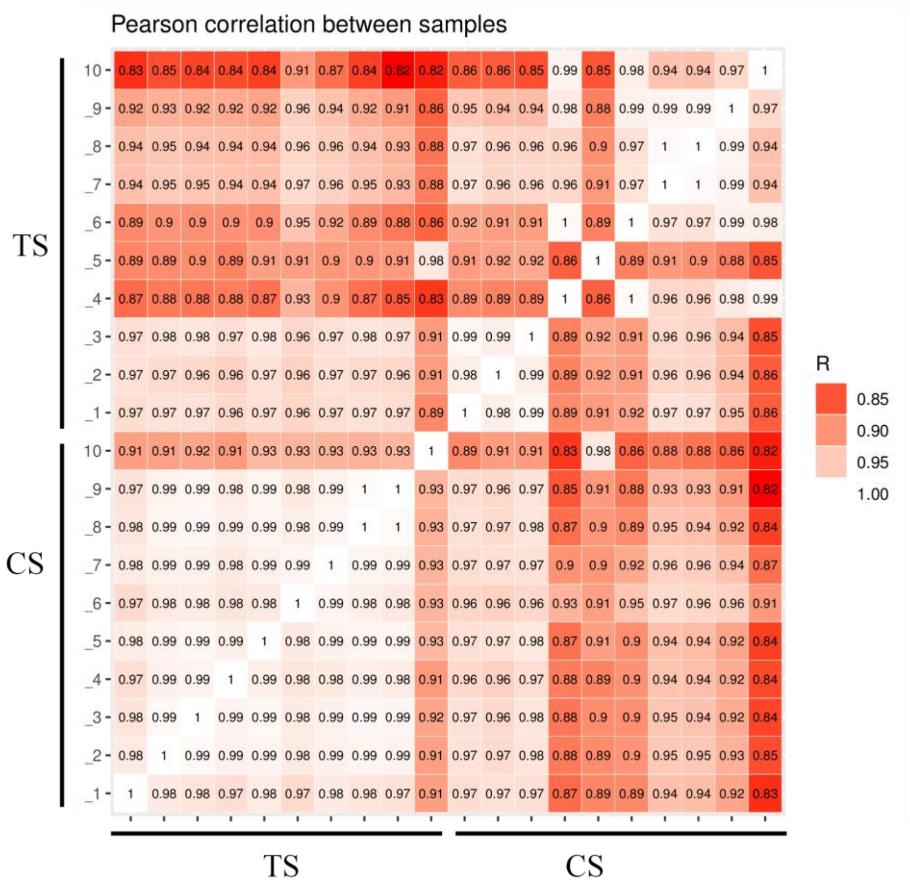

Figure S2 **Correlation coefficient analysis of the data from TS and CS sample groups.**

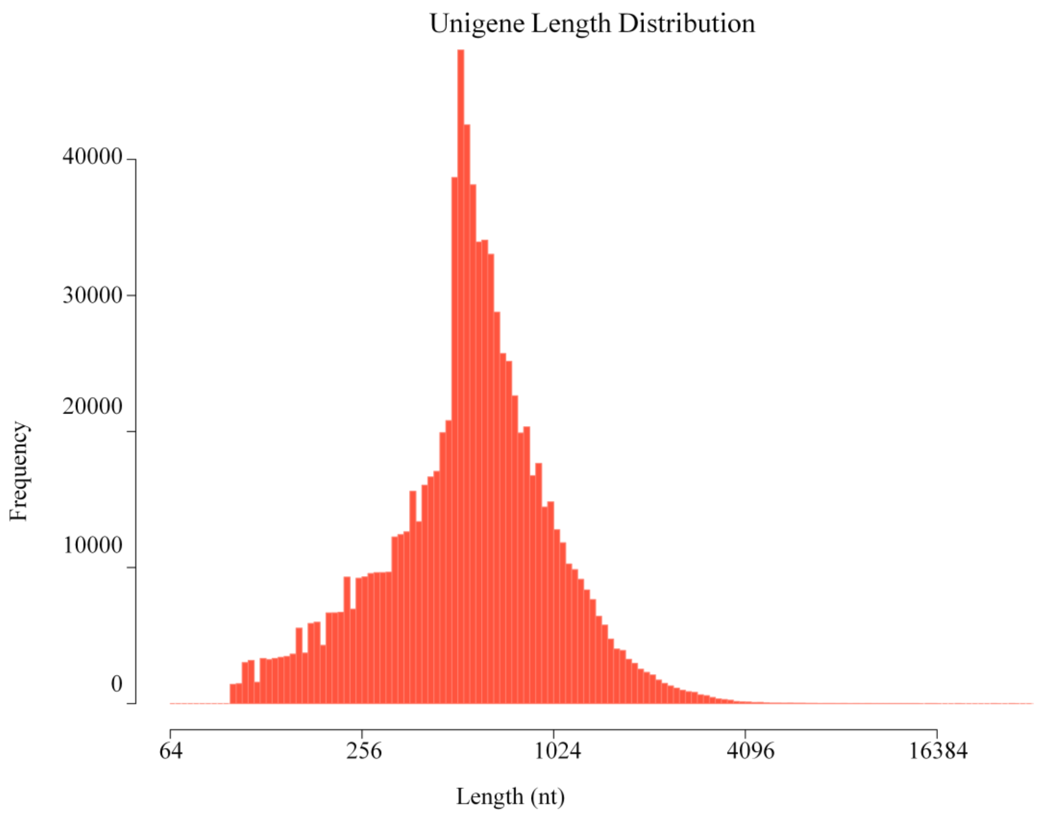

Figure S3 **Length distribution of all predicted unigenes.**

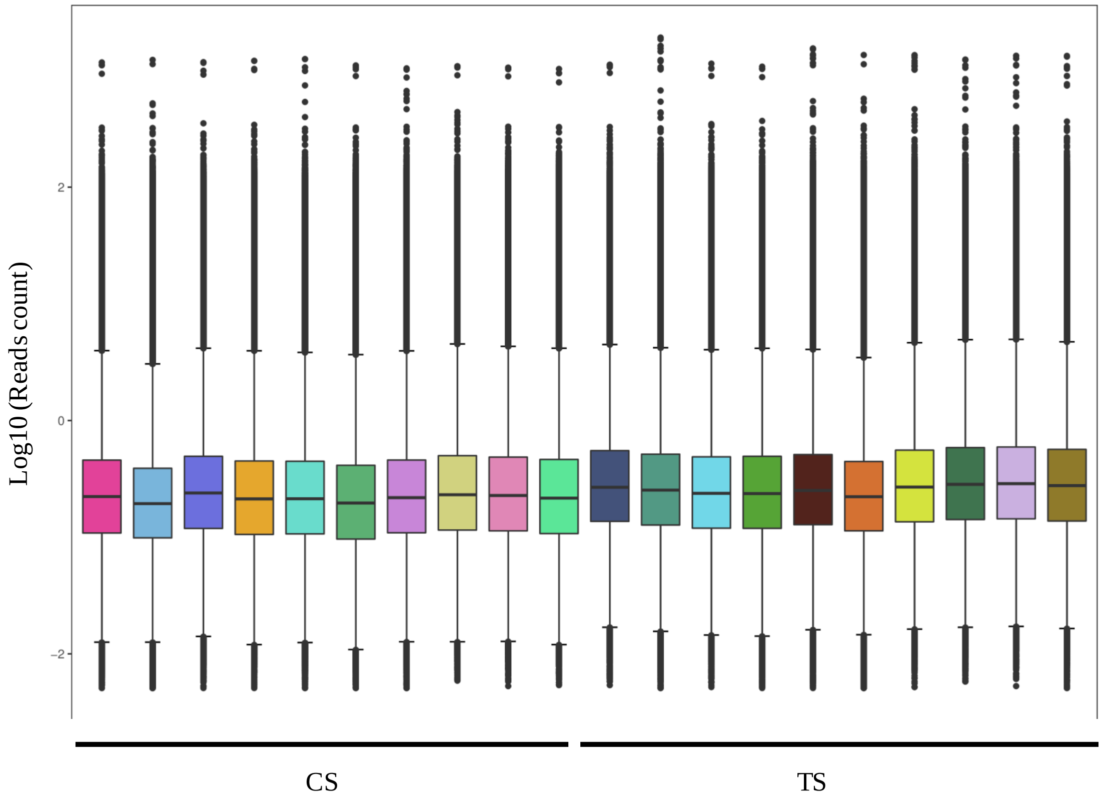

Figure S4 **The read counts of all the predicted unigenes**.

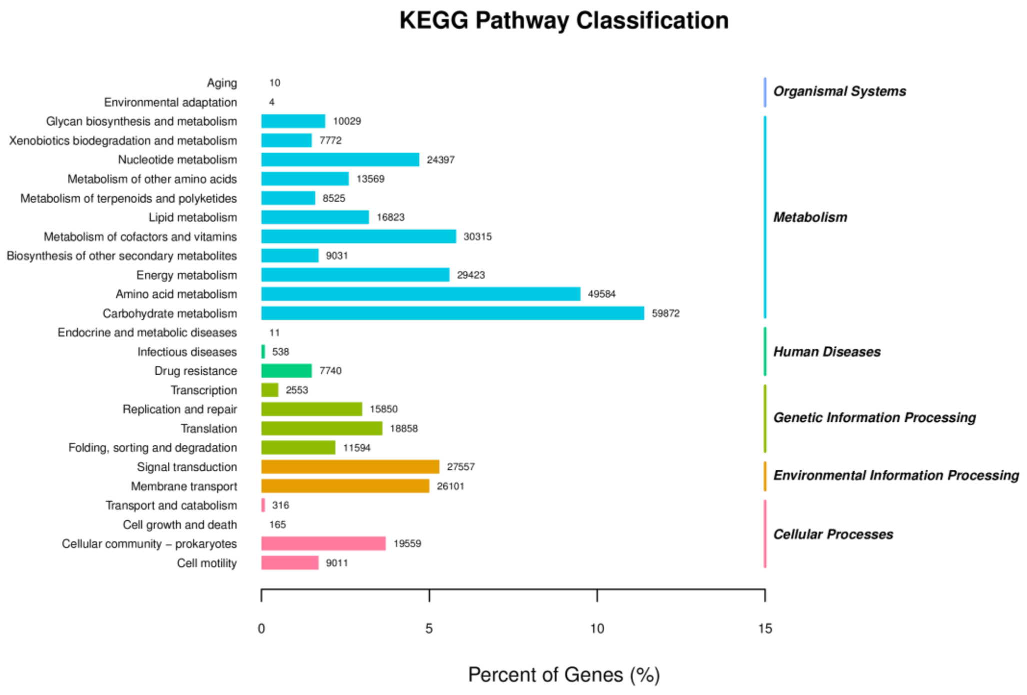

Figure S5 **KEGG analysis of all the predicted unigenes.**

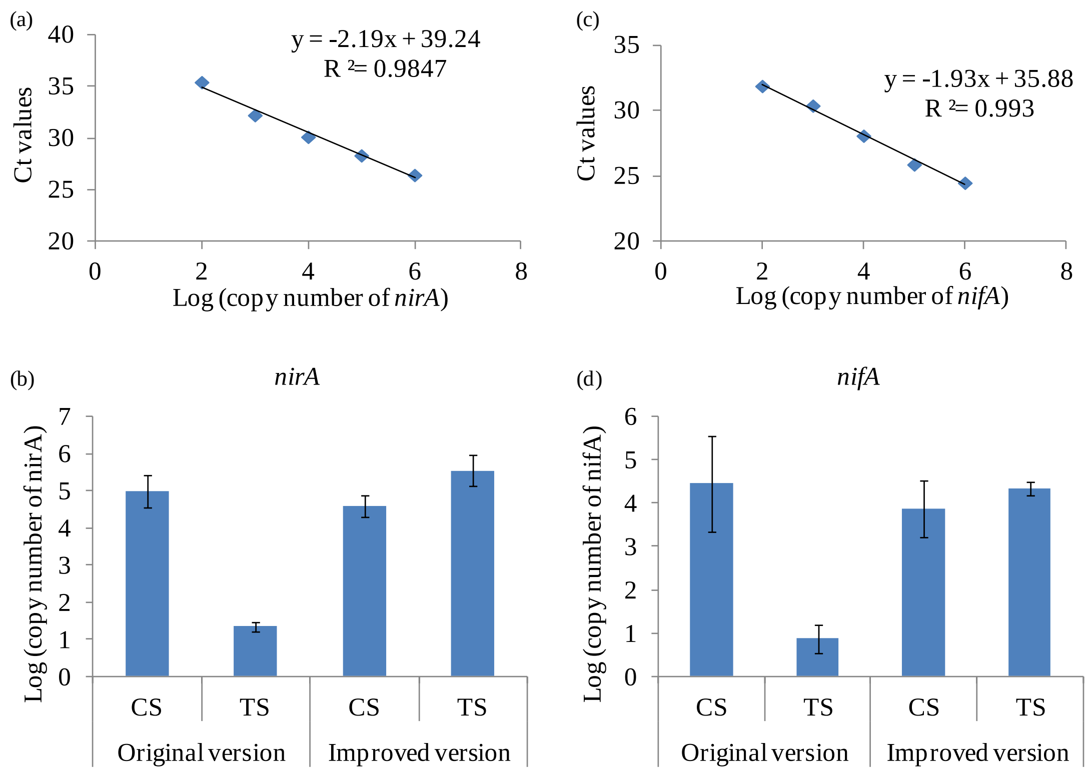

Figure S6 **Validation of two key genes involved in the nitrogen metabolic pathways.** (a) Standard curves of the copy number of *nirA* gene. (b) The copy number of *nirA* gene. (c) Standard curves of the copy number of *nifA* gene. (d) The copy number of *nifA* gene. CS: control sample; TS: treatment sample.

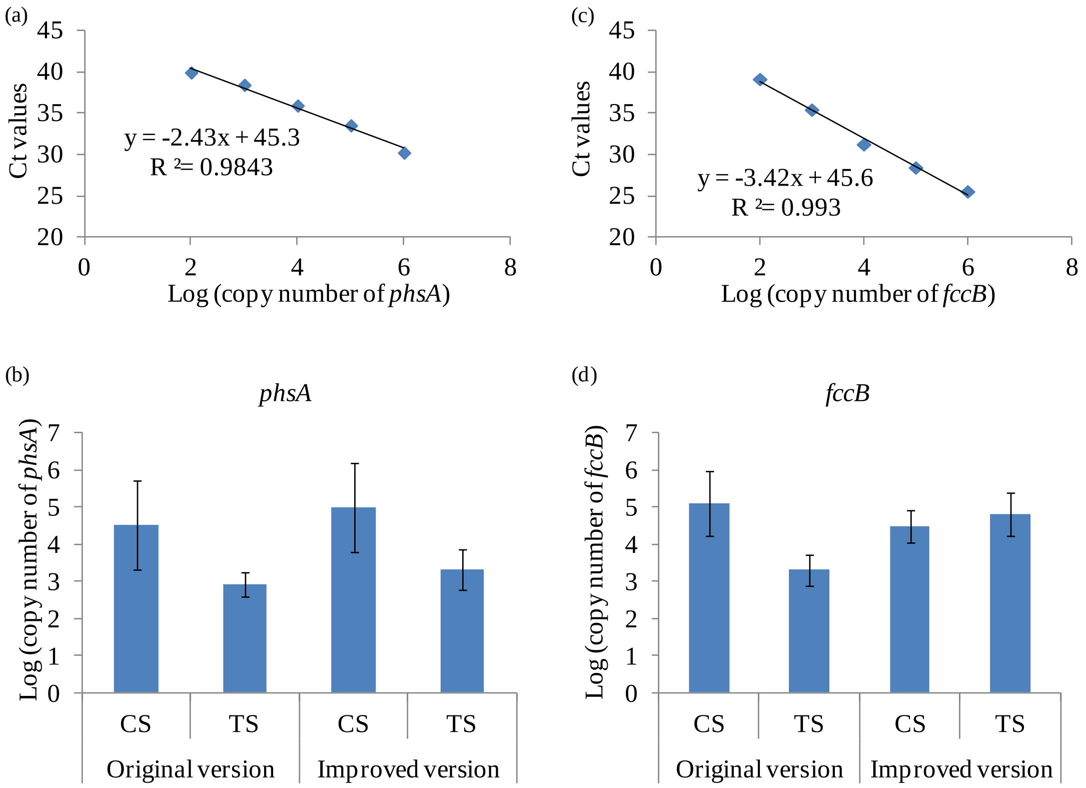

Figure S7 **Validation of two key genes involved in the sulfur metabolic pathways.** (a) Standard curves of the copy number of *phsA* gene. (b) The copy number of *phsA* gene. (c) Standard curves of the copy number of *fccB* gene. (d) The copy number of *fccB* gene. CS: control sample; TS: treatment sample.
